# Current News

**Published:** 1891-02

**Authors:** 


### Current News.

---

At the Annual Meeting of the New York Odontological Society, held at the New York Academy of Medicine, No. 17 West Forty-third Street, Tuesday evening, December 16, 1890, the following officers were elected for the year 1891:

President, Wm. H. Dwinelle; Vice-President, Albert H. Brockway; Treasurer, Dr. Charles Miller; Recording Secretary, Dr. F. A. Remington; Corresponding Secretary, Dr. George A. Wilson; Curator, Dr. J. G. Sailer.

These officers, together with an Executive Committee and Editor (to be chosen by the above-named officers), will constitute the council of the Society.

---

Northern Ohio Dental Association.—The Thirty-second Annual Meeting will be held in Oberlin, Ohio, at the Young Men's Christian Association, Association Building, Tuesday, May 12, 1891, at ten o'clock a.m., and continue its sessions three days. A cordial invitation is extended to all the profession.

Henry Barnes, *Corresponding Secretary.*

F. S. Whitslar, *President.*

TAKING AN IMPRESSION WITH COMPOSITION.—First of all, I detach the handle from the tray and fix it in a slot, made so that it can be easily withdrawn. Having softened the composition, smeared well with vaseline, it is held over the lamp for a moment, and then placed in position in the mouth. The handle is then withdrawn, plenty of cold water used to gargle the mouth, then handle replaced and tray withdrawn. Result, a very perfect model.

A. BURNE, D.D.S.

SYDNEY, AUSTRALIA.

---

### UNITED STATES PATENT-OFFICE.

### DENTAL ANODYNE.

*Specification forming part of Letters Patent No. 394,693, dated December 18, 1888.*

*To all whom it may concern:*

Be it known that I, Robert Isaac Hunter, of Norfolk, in the county of Norfolk and State of Virginia, have invented a new and useful improvement in Dental Anodynes, of which the following is a specification.

My invention is an improvement in anodynes or obtundents employed for allaying the sensitiveness of decayed teeth, and thereby preventing pain during the operation of excavating the cavities preparatory to filling.

My compound is composed of the following ingredients, in the proportions stated: Chloral hydrate, six grains; cocaine, five grains; *arsenic, ten grains;* creosote, twenty drops; carbolic acid, five drops. These substances are put together and three drachms of water are added to form a complete solution of the solid matters. . . .

What I claim is,—

The improved dental obtundent or anodyne hereinbefore described, composed of chloral, six grains; cocaine, five grains; arsenic, ten grains; creosote, twenty drops, and carbolic acid, five drops, substantially as specified.

ROBERT ISAAC HUNTER.

Witnesses:

AMOS W. HART,

D. L. HAZARD.

### DR. THOMAS W. EVANS.

THE *New York Tribune*, of December 28, has a very interesting article of nearly two columns, giving a history of the life-work of this gentleman. The following extract contains information not generally known, and which will recall to older dentists the excellent early training of Dr. Evans in the well-remembered place in Merchant Street, Philadelphia, where dentists of the earlier day procured their gold and silver solder, and physicians the most approved pessaries and delicate instruments.

"The doctor was called in as a friend when the Crown Prince Frederick was taken to San Remo, and remained there during the prince's stay. I happened to be at San Remo at the time, and it was there I became really well acquainted with the famous doctor. Every day he received despatches from the Empress Augusta at Berlin. It was he who sent her private advices constantly of the condition of her son upon the terrible day when it was thought that the Crown Prince would strangle when the operation of tracheotomy was decided upon. There was no one in the little Italian village skilful enough to make the silver tube necessary to be used after the operation was performed. It was here the skilled hand of the American doctor was called into play. I walked down with him to a little jeweller's office in San Remo, and saw him put on his workman's apron and begin with a blow-pipe and a hammer upon a five-franc piece. He worked there all night, and the next morning a beautifully-made silver tube was ready, and the life of the prince was prolonged, where suffocation would probably have set in within the next twenty-four hours. There was no mention made of Dr. Evans in the story of this operation. In the English papers it was Dr. Morell Mackenzie who did everything, even to the making of the silver tube which no one but an extremely skilful man, with a natural turn for mechanics, could have made with the simple materials found in a country jeweller's shop."

